# Progress in the performance of HIV early infant diagnosis services in Zambia using routinely collected data from 2006 to 2016

**DOI:** 10.1186/s12889-018-6222-y

**Published:** 2018-11-26

**Authors:** Jasleen Singh, Suzanne Filteau, Jim Todd, Sehlulekile Gumede-Moyo

**Affiliations:** 1 Department of Population Health, Faculty of Epidemiology and Population Health, London School of Hygiene and Tropical Medicine, Keppel Street, London, WC1E 7HT UK; 2 School of Public Health, University of Zambia, Lusaka, Zambia

**Keywords:** Early infant diagnosis, HIV, PMTCT

## Abstract

**Background:**

Early diagnosis and treatment initiation of HIV-infected infants can greatly reduce the risk of infant mortality. The WHO recommends testing HIV-exposed infants at 6 weeks of age and immediate initiation of antiretroviral therapy if positive. This study aimed to determine the feasibility of using an electronic health records system to evaluate the performance of Zambia’s HIV Early Infant Diagnosis services.

**Methods:**

A retrospective analysis of routinely collected data from the Zambian SmartCare database was performed for the period January 2006 to December 2016. The study population includes all HIV-infected infants (*n* = 32,593) registered during this period on treatment for HIV. Univariable logistic regression was conducted to identify factors associated with later infant testing and treatment initiation.

**Results:**

The mean age at infant HIV test decreased from 10.10 months in 2006 to 3.49 months in 2016. Infants born in 2015 were almost 4 times more likely to be tested under 2 months of age compared to infants born in 2006 (OR: 3.72, *p*-value: < 0.001). The mean time from diagnosis to treatment initiation decreased from 220 days in 2006 to 9 days in 2015. There was substantial regional variability with infants in the provinces of Copperbelt, Luapula and Southern performing best in outcomes and Eastern, Lusaka and Western performing the worst.

**Conclusions:**

HIV-exposed infants born more recently have significantly better outcomes than infants born a decade ago in Zambia, which could be as a result of increased attention and funding for HIV programmes.

## Background

Early diagnosis and treatment initiation of HIV-infected infants with antiretroviral therapy (ART) can reduce the risk of early infant mortality by 76% [1]. However, treatment for HIV-infected children lags considerably behind adult treatment and without treatment approximately 50% of HIV-infected infants die before the age of two [2]. The World Health Organisation (WHO) recommends testing HIV-exposed infants by 6 weeks of age and immediate initiation of ART if positive. Despite these recommendations infants are lost at every step of the early infant diagnosis cascade [3].

Zambia has been using the electronic health records system SmartCare for the routine collection of HIV data since 2004. SmartCare was developed to improve continuity of care and provide timely data on maternal and child health HIV interventions for public health purposes. To date no analysis of the paediatric HIV data has been performed. This study was a retrospective analysis of routinely collected data from the SmartCare database over the period 2006 to 2016, to determine if infants are being tested and initiated on treatment in the correct timeframe. This information can be used to inform decisions on improving the provision of early infant diagnosis services in Zambia.

## Methods

### Study design

This retrospective analysis of routinely collected data was conducted as part of the SEARCH (Sustainable Evaluation through the Analysis of Routinely Collected HIV data) project which aims to support the utilisation of routinely collected HIV data. The SEARCH project is collaboration between the London School of Hygiene and Tropical Medicine and the Ministries of Health in Zambia and Tanzania. The data source was SmartCare, one of the largest electronic patient monitoring systems in Africa. Introduced as a pilot project in 2004 by the Zambian Ministry of Health with funding from the US Centers for Disease Control (CDC), it has now been rolled out across all ten provinces of Zambia and is used to monitor and plan improvements in the country’s HIV services. All facilities in Zambia wishing to dispense ART are required to use SmartCare. Since 2005 SmartCare has been deployed to over 800 facilities in 94 districts, with an enrolment above 900,000 patients. This represents approximately 40% of all clinics in Zambia; these are the largest and busiest ART clinics and the requirement to join SmartCare if clinics wish to prescribe ART means that most Zambian patients on ART care captured in SmartCare databases [4].

Data was extracted by a SEARCH team member from the Zambian SmartCare database modules, Paediatric ART and Under 5 Registration, between the years 2006 and 2016 using a standardised data extraction form. The study population includes all HIV-infected infants registered during this period on treatment for HIV. Age at infant HIV test was determined by linking the Under 5 Registration module (which has infant date of birth) and Paediatric ART module (which has date of infant HIV test). Infants are registered as independent patients from their mothers and the system does not have a link between the mother and infant pairs; therefore, information could not be collected on the mother’s treatment, on the proportion of HIV-exposed infants who tested HIV-negative, or on HIV-exposed infants who missed both testing and provision of ART. For this reason, we are focusing this analysis solely on HIV-infected infants born between January 2006 and December 2016 who have received ART.

### Statistical methods

Categorical variables were summarised by frequencies and percentages and continuous variables by histograms. Univariable and multivariable logistic regression were conducted with “age at HIV test” and “time from diagnosis to treatment initiation” as the dependent variables and the following independent variables: infant sex, province, year of birth, and season of birth. Season were defined as early dry (June to August); late dry (September to November); early rainy (December to February) and late rainy (March to May).

Odds ratios with 95% confidence intervals were calculated to identify risk factors for age at test under 2 months and time from diagnosis to treatment under 2 weeks. For the variable ‘province’ Lusaka was chosen as the reference group because it is the most populous province, and it contains the capital city where the SmartCare project was first rolled out so has the largest number of registered infants. Although multivariable analysis was also conducted for all variables to display adjusted odds ratios, the results showed little difference and so only the univariable analysis results are included in this paper. All analysis was performed using STATA 14 and graphics were produced using STATA and Microsoft Excel.

## Results

A total of 32,593 HIV-infected infants on ART in Zambia were identified from SmartCare over the period 2006 to 2016. The number of infants in the database on ART increased from 1761 in 2006 to peak at 3720 in 2009 and then steadily decreased to 108 in 2016. Main comparisons over time used 2015 since there were too few infants listed in 2016 for age at testing and none for time to treatment initiation.

### Age of diagnosis

**Fig. 1:**
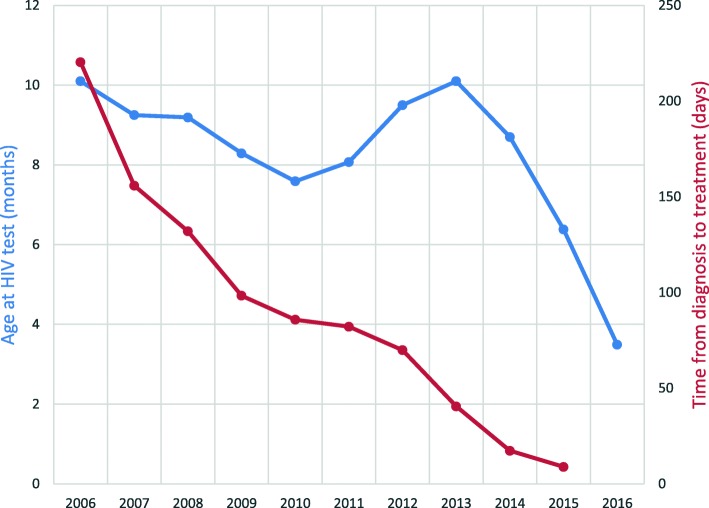
Mean age at HIV test and time from diagnosis to treatment initiation by year of birth. Blue line is Age at HIV Test and the Red line is the Time from diagnosis to treatment initiation

For the outcome ‘age at infant HIV testing’, 20,260 (62.16%) infants had complete data recorded. Looking at the country as a whole, the mean age at HIV testing has steadily decreased from 10.10 months for infants born in 2006 to 3.49 months in 2016 (Fig. 1). Infants born in 2015 were more likely to be tested under 2 months of age compared to infants born in 2006 (OR: 3.72, *p*-value: < 0.001). For infants born in 2016 an even greater association was found (OR: 5.62, *p*-value: < 0.001), but this result must be viewed with some caution as there were far fewer infants in the database for the year 2016 (*n* = 108) than 2015 (*n* = 386).

**Fig. 2:**
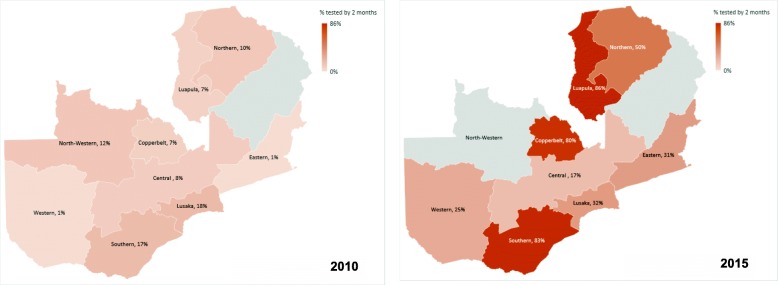
Percentage of HIV tests performed within 2 months of birth by province in 2010 and 2015.The percentages represent the proportion of infants who were tested within 2 months of birth by province in 2010 and 2015.NB//There was no 2015 data for North-Western province. The other missing province is Muchinga province which was newly created in 2011 and is not included in this database

Considering all years together, infants in all provinces, especially Western province (OR: 0.21, *p*-value: < 0.001) were less likely to be tested under 2 months of age as compared to infants in Lusaka; an exception was Southern province which had an increased odds (OR: 1.83, p-value: 0.001). Figure 2 shows the percentage of HIV tests performed within 2 months of birth in the years 2010 and 2015 by province. All provinces show an overall improvement, particularly Copperbelt, Luapula and Southern. The years 2010 to 2015 were chosen for the comparison to coincide with the changes in prevention of mother-to-child transmission (PMTCT) guidelines in Zambia over this period, as discussed below.

Infants born in the late dry season were more likely to be tested within 2 months than infants born in the early dry season (OR 1.39; *p* value: < 0.001) (Table 1). There was no association found for sex.

**Table 1:** Univariable analysis results for sex, season, province and year of birth

Variable	Age at test	Time from diagnosis to ART	Logistic regression for testing at < 2 months			Logistic regression for time from diagnosis to ART < 2 weeks		
	< 2 months n (%)	< 2 weeks n (%)	OR	95% CI	*P*-value	OR	95% CI	*P*-value
*Sex*								
Male	550/10,537 (5.2)	2249/10,537 (21.3)	1.00	–	–	1.00	–	–
Female	603/11,185 (5.4)	2568/11,158 (23.0)	1.03	0.92, 1.17	0.573	1.10	1.03, 1.17	0.004
*Season*								
Early dry	914/5254 (17.4)	1478/5254 (28.1)	1.00	–	–	1.00	–	–
Late dry	946/4168 (22.7)	1135/4168 (27.2)	1.39	1.26, 1.54	< 0.001	0.96	0.87, 1.05	0.333
Early rainy	926/5364 (17.3)	1509/5364 (28.1)	0.99	0.90, 1.10	0.856	1.00	0.92, 1.09	0.999
Late rainy	912/5474 (16.7)	1611/5474 (29.4)	0.95	0.86, 1.05	0.311	1.07	0.98, 1.16	0.137
*Province*								
Lusaka	365/5394 (6.8)	822/5394 (15.2)	1.00	–	–	1.00	–	–
Central	94/2288 (4.1)	688/2288 (30.1)	0.59	0.47, 0.74	< 0.001	2.39	2.13, 2.69	< 0.001
Copperbelt	294/4778 (6.2)	1648/4778 (34.5)	0.90	0.77, 1.06	0.210	2.93	2.66, 3.22	< 0.001
Eastern	59/2072 (2.8)	431/2072 (20.8)	0.40	0.31, 0.53	< 0.001	1.46	1.28, 1.66	< 0.001
Luapula	56/1341 (4.2)	547/1341 (40.8)	0.60	0.45, 0.80	0.001	3.83	3.36, 4.37	< 0.001
Northern	57/983 (5.8)	350/983 (35.6)	0.85	0.64, 1.13	0.262	3.08	2.65, 3.57	< 0.001
North-Western	25/883 (2.8)	244/883 (27.6)	0.40	0.27, 0.61	< 0.001	2.12	1.80, 2.50	< 0.001
Southern	394/3366 (11.7)	718/3366 (21.3)	1.83	1.57, 2.12	< 0.001	1.51	1.35, 1.69	< 0.001
Western	24/1577 (1.5)	285/1577 (18.1)	0.21	0.14, 0.32	< 0.001	1.27	1.06, 1.42	0.007
*Year of birth*								
2006	220/1709 (12.9)	340/1709 (19.9)	1.00	–	–	1.00	–	–
2007	412/2619 (15.7)	547/2619 (20.9)	1.26	1.06, 1.51	0.009	1.06	0.91, 1.24	0.430
2008	528/3475 (15.2)	790/3475 (22.7)	1.21	1.02, 1.44	0.025	1.18	1.03, 1.37	0.020
2009	683/3678 (18.6)	861/3678 (23.4)	1.54	1.31, 1.82	< 0.001	1.23	1.07, 1.42	0.004
2010	675/2718 (24.8)	726/2718 (26.7)	2.24	1.89, 2.65	< 0.001	1.47	1.27, 1.70	< 0.001
2011	446/1812 (24.6)	612/1812 (33.8)	2.21	1.85, 2.64	< 0.001	2.05	1.76, 2.40	< 0.001
2012	191/1384 (13.8)	564/1384 (40.8)	1.08	0.88, 1.33	0.450	2.77	2.35, 3.26	< 0.001
2013	186/1352 (13.8)	605/1352 (44.7)	1.08	0.88, 1.33	0.474	3.26	2.76, 3.85	< 0.001
2014	171/1019 (16.8)	548/1019 (53.8)	1.36	1.10, 1.70	0.005	4.68	3.90, 5.62	< 0.001
2015	137/386 (35.5)	140/386 (36.3)	3.72	2.87, 4.83	< 0.001	2.29	1.80, 2.92	< 0.001
2016	49/108 (45.4)	0/108 (0.0)	5.62	3.71, 8.51	< 0.001	–	–	–

### Time to ART initiation

For the outcome ‘time from diagnosis to ART initiation’, 10,881 (33.38%) infants had complete data recorded. At the country level the mean time from diagnosis to starting treatment has decreased significantly from 220 days for infants born in 2006 to 9 days in 2015 (Fig. 1). Infants born in 2015 were more likely to start treatment in under 2 weeks compared to infants born in 2006 (OR 2.29; *p* value: < 0.001).

Infants in all provinces had an increased likelihood of starting treatment within 2 weeks as compared to Lusaka, despite being less likely to be tested under 2 months. Luapula showed the biggest difference (OR: 3.83, *p*-value: < 0.001). The provinces showing the most improvement from 2006 to 2015 were Copperbelt, Central and Northern. Female infants were slightly more likely to start treatment in under 2 weeks compared to males (OR: 1.10; *p* value: 0.004). Season showed no significant association.

## Discussion

In Zambia the age at infant HIV test has shown a steady decline, with the exception of the period 2010 to 2013 which showed a slight increase. This period coincides with the implementation of new WHO treatment guidelines for PMTCT. Changes in WHO recommendations over the past decade on when to initiate ART in pregnant women have had major implications for the delivery of HIV services, and help explain the trends in service delivery. From 2010 Zambia adopted Option A, a complex guideline consisting of different maternal treatment regimens during pregnancy, labour and postpartum as well as infant prophylaxis [5]. This complexity of regimen changes, combined with the need for regular clinic visits in early infancy, led to high attrition rates and infants receiving improper doses of daily Nevirapine [6]. Option B+ which was adopted in 2013 [7] removed this barrier by recommending treating all pregnant women with lifelong ART regardless of CD4 count. By simplifying the process considerably, in Malawi (where it was first trialled), switching to Option B+ led to a five-fold increase in the number of pregnant women enrolled on ART in the first quarter of implementation [8]. When Zambia announced its policy for universal ART in 2015, there was an increase in the volume of patients initiating on ART [9]. Zambia has made progress towards elimination of HIV mother-to-child transmission with a reduction in new HIV infections among children from 10,000 in 2010 to 8900 in 2016. This could be as result of improved coverage of pregnant women living with HIV accessing antiretroviral medicines to 86% [10]. In our study we were not able to calculate the MTCT rate because the SmartCare database does not have information on the HIV exposed infants who are not on ART.

All provinces showed a subsequent reduction in age at test between 2013 and 2015 with the implementation of Option B+, in which all pregnant women with HIV are offered lifelong ART regardless of CD4 count [11]. The progress could also be attributed to Option B+ as previous studies have concluded that children born to women who received ART are less likely to be lost to follow-up and more likely to be tested for HIV [12–14]. This suggests that under Option B+ mothers and their infants are more likely to be attached to the health care system. Therefore efforts have to be made to ensure that the infants who are tested and initiated are retained in care. In Tanzania, 61% of infants receiving treatment were lost to follow up at the time of review, despite the high proportion of guardians and parents who returned for PCR results (92% in 2010 and 98% in 2011) [15]. The results were consistent with a study from Malawi were 48% of the HIV-exposed infants were declared lost to follow up (LTFU) in the database although 96% of the them in the cohort had their PCR test done at 24 months [16]. Hence despite the reduction in the age of testing the progress of EID must be enhanced by ensuring continuity in care. In Zambia the estimated percentage of children (aged 0–14 years) living with HIV receiving ART, in 2015 was 61% [17], 3% lower than the adult coverage.

An effective EID service should achieve the following: identify all HIV-exposed infants, provide HIV testing and ensure return of results in a timely manner; retain HIV-exposed infants and their mothers in care; and identify all HIV-infected infants and link them to treatment services to ensure timely initiation of ART [18]. Our data set could not allow us to effectively analyse all these steps hence our conclusion of the progress of EID could have been overestimated as only 33.38% infants had complete data recorded. The poor data quality might have an effect on the external validity of the study. Hence the reasons for poor data quality have been qualitatively explored (*Gumede-Moyo* et al. submitted). It is also likely that poor data entry over these years has impacted the results; SmartCare has evolved from a system solely used to track patients into an extensive database of all patients receiving ART in the country. This has resulted in a large amount of data being collected for each patient which is very time consuming for the clinician, who often omits collecting data for certain fields they deem irrelevant to their patient’s care. In addition, power outages in remote areas of Zambia are a major problem and can occur for prolonged periods of time during the day, limiting the time that data can be entered into the system. This means that data collection is often not up to date and this could contribute to the apparently poor performance of Western province.

Our study is the first to attempt to analyse the progress of EID using the SmartCare database as a source for exposed infants on ART dataset. A more comprehensive dataset of the HIV status of all exposed infants could have been obtained from the ANC and Under 5 paper registers. However, the majority of studies using paper based routine data have also acknowledged the common problem of missing data [19]. This could be assumed to be one of the causes of under-utilisation of routinely collected data which was observed by Munthali et al. [20].

The best performing provinces in 2015 (Fig. 2) for ‘percentage of tests performed within 2 months of birth’ were Luapula, Southern and Copperbelt; surprisingly Lusaka was the second worse performing province after Western. Of the nine provinces of Zambia (excluding Muchinga province which was created in 2011 and not included in this database), Lusaka and Copperbelt are predominantly urban whereas the remaining are predominantly rural [21]. Previous studies have shown that the burden of HIV is highest in Zambia’s ‘urban poor’ [22, 23], and indeed data from the latest Zambian 2013–14 DHS showed that Lusaka and Copperbelt have the highest adult HIV prevalence, at 16.3 and 18.2% respectively [24]. Despite the similar HIV prevalence and population sizes, Copperbelt saw a more dramatic improvement in age at infant testing between 2013 and 2015 than Lusaka. The stark difference between the performance of Copperbelt and Lusaka is also seen in the percentage of infants tested under 2 months of age, which was amongst the highest in Copperbelt in 2015 compared to 2010, but rose much less in Lusaka during the same period. This regional variability seen could be attributed to the concentration of donor funded programmes in the various provinces. Despite Zambia’s classification as a lower-middle income country, it remains heavily dependent on external donors to finance its national HIV response. PEPFAR and the Global Fund account for 95% of donor funding for HIV care [25]. Given external aid makes up the bulk of HIV funding in Zambia, it is possible that decisions on which areas of the country to target may influence the trends in HIV outcomes we have seen at a provincial level.

Infants born in the early dry season tended to be tested earlier compared to infants born in the late dry season, although there was no association found between dry and rainy seasons. Season was chosen as a variable of interest because previous studies in Sub-Saharan Africa have shown an association between being born in the rainy season and poorer outcomes for HIV-exposed infants [26, 27]. Reasons suggested for the higher mortality experienced by infants born in the rainy season include a more contaminated water supply from the rains and food scarcity in the period preceding the harvest [28, 29].

### Study limitations

This research has shown that routinely collected data offers a valuable opportunity for the near real-time surveillance of large quantities of data. The SmartCare database of registered infants receiving ART represents a robust sample of the population under study over the stated time period, although we recognise there was a steady decrease in the numbers of infants registered onto SmartCare from 2009 onwards. The large amounts of missing data did not only introduce bias but also compromised the statistical power of a study. Other limitations with using this database are that the database was designed for practical use by healthcare workers and not research so the variables collected are limited to those deemed most useful for the clinical care of patients. In addition, mother-infant pairs are not linked within SmartCare, and so the analysis was restricted to HIV-infected infants on ART and we were not able to analyse outcomes for HIV-exposed uninfected infants. This would be valuable information for any future assessment of mother-to-child HIV transmission prevention and control programs in Zambia.

## Conclusions

Early infant diagnosis of HIV is essential to achieve prompt treatment initiation and reduce infant mortality. Infants born more recently have better clinical HIV care than infants born a decade ago in Zambia, which could be as a result of more inclusive treatment eligibility guidelines. Provincial variability in the performance of early infant diagnosis services is substantial. Further research is needed on the reasons for such stark regional disparities in HIV service provision in Zambia, and on addressing missed opportunities for infant testing.

sation.

## Abbreviations

AIDS: Acquired immune deficiency syndrome
ART: Antiretroviral therapy
CD4: T-lymphocyte cell bearing CD4 receptor
CDC: Centres for disease control and prevention
DHS: Demographic and Health Survey
EID: Early infant diagnosis
HIV: Human immunodeficiency virus
LSHTM: London School of Hygiene and Tropical Medicine
LTFU: Lost to follow up
MTCT: Mother-to-child transmission
PEPFAR: President’s emergency plan for AIDS relief
PMTCT: Prevention of mother-to-child transmission of HIV
SEARCH: Sustainable Evaluation through the Analysis of Routinely Collected HIV data
UNAIDS: Joint United Nations Program on AIDS
WHO: World Health Organisation
